# Continuous Bone–Quadriceps Tendon–Rectus Femoris Autograft for Revision Anterior Cruciate Ligament and Anterolateral Ligament Reconstructions

**DOI:** 10.1016/j.eats.2025.103985

**Published:** 2025-10-31

**Authors:** Vincent Morin, Guillaume Veyrat, Enora Pennec, Benoit Gaulin, Pierre Girard, Matthieu Ollivier, Clément Horteur

**Affiliations:** a Hôpital Privé Médipole de Savoie, Challes-les-Eaux, France; b Département de Chirurgie Orthopédique et Traumatologie du Sport, Hôpital Sud, CHU Grenoble Alpes, Échirolles, France; c Department of Orthopaedics and Traumatology, Institute for Locomotion, Aix Marseille Univ, APHM, CNRS, ISM, Sainte-Marguerite Hospital, Marseille, France

## Abstract

Revision anterior cruciate ligament (ACL) reconstruction is a frequent procedure due to the significant incidence of rupture following primary reconstruction. Achieving strong graft fixation alongside favorable biological conditions is essential for optimal graft healing, which can present challenges in the context of revision surgery. Additionally, the addition of anterolateral ligament reconstruction is recommended during revision procedures, necessitating adequate graft length or the use of a secondary harvest site. Consequently, the selection of an appropriate autograft for revision ACL reconstruction is critical to satisfy these requirements. Recently, the rectus femoris tendon and its aponeurosis have been proposed as viable autograft options for ACL reconstruction. Herein, we describe the surgical technique for combined ACL and anterolateral ligament reconstruction using a continuous autograft composed of a patellar bone block, partial-thickness quadriceps tendon, and the rectus femoris tendon and aponeurosis.

Anterior cruciate ligament (ACL) injuries are common in athletic populations, and their incidence has doubled over the past 2 decades.^1^^,^^2^ Surgical reconstruction remains the gold standard for restoring knee stability and enabling return to sport.^3^

To date, autologous grafts such as bone–patellar tendon–bone, hamstring tendons, and quadriceps tendon (QT) are the most widely used,^4^ with each presenting specific advantages and limitations.^5^, ^6^, ^7^, ^8^ In recent years, the quadriceps tendon has gained increasing popularity due to its consistent size and favorable biomechanical properties, in both primary and revision ACL reconstructions.^9^, ^10^, ^11^, ^12^

The quadriceps tendon is composed of multiple muscular contributions, including the vastus medialis, vastus lateralis, vastus intermedius, and rectus femoris (RF), with the latter providing longitudinal fibers particularly suited for graft preparation.^13^, ^14^, ^15^ While most QT grafts are harvested in full-thickness fashion (or respecting its deep layer), recent studies have proposed the isolated use of the rectus femoris tendon and aponeurosis for ACL reconstruction.^16^, ^17^, ^18^, ^19^ Huber et al.^20^^,^^21^ showed sufficient length and diameter for intra-articular reconstruction, with comparable outcomes to hamstring grafts in the revision setting. Similarly, Rego et al.^22^ and Sonnery-Cottet et al.^23^ showed that the RF tendon could be effectively used for combined ACL and anterolateral ligament reconstruction. Furthermore, Fink et al.^24^ proposed a minimally invasive technique for harvesting QT grafts with or without a bone block, paving the way for tissue-sparing approaches. However, compared to pure rectus femoris autograft, tibial graft fixation and graft quality can be improved when harvesting a patellar bone block and the quadriceps tendon, along with the rectus femoris.^22^^,^^23^

In this Technical Note, we present a technique for revision ACL reconstruction using a composite autograft composed of the rectus femoris tendon in continuity with a partial-thickness quadriceps tendon and a patellar bone block. We detail the surgical procedure and discuss its potential advantages over conventional grafts, including its adaptability for combined intra- and extra-articular reconstructions.

## Surgical Technique

**Fig 1:**
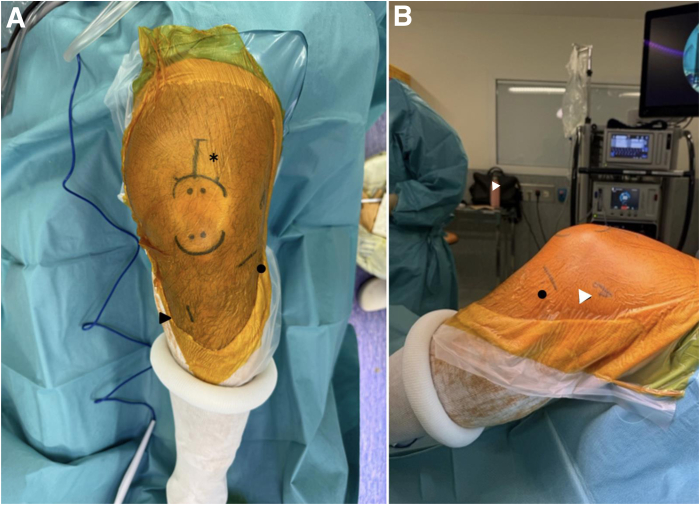
The surgical setup features a left knee flexed to 80°, patient in the supine position. The anterior (A) and lateral (B) perspectives. Incision sites have been delineated using a dermographic pen as follows: the graft harvest region adjacent to the proximal patella (indicated by an asterisk), the tibial tunnel (black arrow), the femoral tunnel (white arrow), and the lateral tibiofemoral joint line (black dot).

A video presenting this technique is provided (Video 1). The procedure is performed under general or locoregional anesthesia with the patient positioned supine, with a pneumatic thigh tourniquet and the knee flexed to 80°. The main anatomic landmarks for performing anterolateral ligament (ALL) reconstruction are marked on the lateral aspect of the knee, including the lateral epicondyle, the joint line, the fibular head, and Gerdy’s tubercle (Fig 1).

### Step 1: Graft Harvesting and Preparation

**Fig 2:**
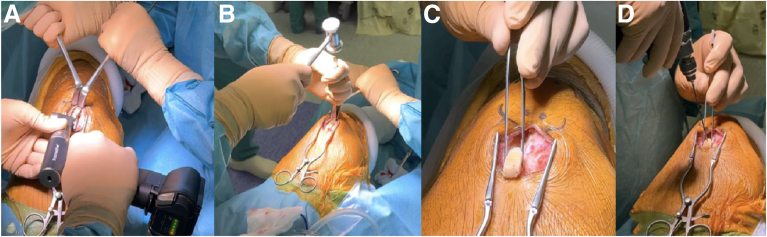
Introperative superior views ( from proximal to distal), harvesting and preparation of the patellar bone block. (A) The bone block, measuring approximately 1 cm in width and 2 cm in length, is delineated and cut using an oscillating saw parallel to the axis of the quadriceps tendon. (B, C) Final detachment is achieved using an osteotome. (D) Two 2-mm transverse holes are then drilled through the bone block to facilitate traction suture placement.

**Fig 3:**
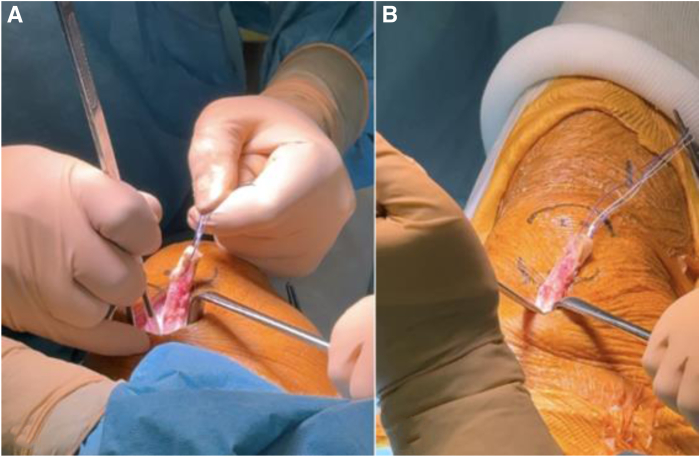
Introperative superior views ( from proximal to distal), harvesting of the quadriceps tendon along with the bone block. With traction applied to the suture placed in the patellar bone block, a 1-cm-wide partial quadriceps tendon autograft—respecting its deep layer (vastus intermedius tendon)—is harvested using a No. 11 surgical blade (A), until an overall length of 8 to 9 cm is obtained (B).

**Fig 4:**
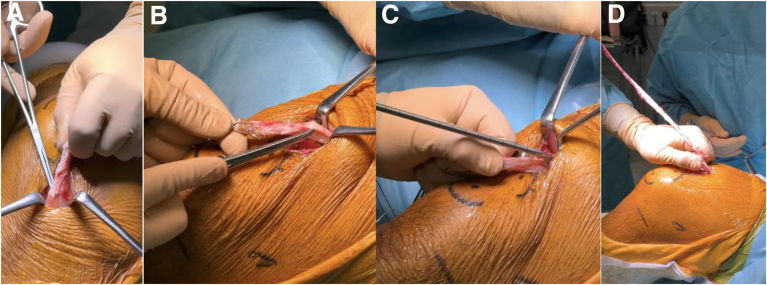
Harvesting of the rectus femoris tendon. Once the quadriceps tendon of the desired length has been obtained, dissection is performed proximally on the graft to identify and isolate the rectus femoris tendon (A), constituting the anterior strand of the quadriceps tendon (B). When sufficient exposure is achieved, an open stripper is positioned (C), and the rectus femoris tendon—along with its aponeurosis—is harvested in a proximal direction, with the stripper guided toward the anterior inferior iliac spine (D).

**Fig 5:**
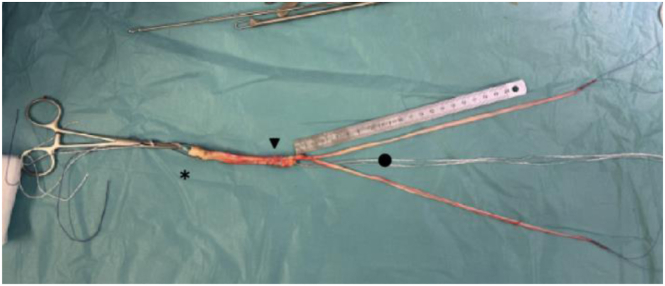
Bone–quadriceps tendon–rectus femoris graft preparation. Once the rectus femoris graft has been cleaned of residual muscle fibers, the graft is split into 2 strands using scissors. For later manipulation of the graft, traction sutures are placed: in the bone block, at the proximal aspect of the quadriceps tendon, and at the end of each rectus femoris strand.

A 4-cm longitudinal skin incision is made along the central aspect of the quadriceps tendon, starting at the superior pole of the patella and extending proximally. After hemostasis of the subcutaneous fat, the quadriceps tendon is exposed. A continuous bone-tendon autograft is harvested from distal to proximal: patellar bone block, partial-thickness quadriceps tendon, and rectus femoris. The bone block is harvested with an oscillating saw (15-20 mm long, 10 mm wide, 8-10 mm thick with 45° angulated longitudinal cuts). Attached to the bone block is a 10-mm-wide and 9-cm-long strand of quadriceps tendon, harvested while preserving its deep layer and 5 mm of tendon medially from the vastus medialis muscular fibers (Fig 2, Fig 4, Fig 3).^25^ The rectus femoris is identified as the superficial layer of the quadriceps tendon and harvested proximally using a stripper (Fig 4). The quadriceps tendon is then detached from its proximal insertion, underneath the rectus femoris. Once harvested, the rectus femoris tendon is split into 2 equal strands showing a “tongue snake” pattern (Fig 5). The free ends of the 2 limbs are whipstitched using resorbable sutures. Nonabsorbable sutures are placed into the bone block. The graft is then soaked for 10 minutes in a sterile compress impregnated with vancomycin solution (2.5 mg/mL; 125 mg/50 mL) for prophylaxis.^26^ The quadriceps tendon defect is closed with interrupted Vicryl 0 sutures (Ethicon).

### Step 2: Diagnostic Arthroscopy and Management of Associated Lesions

Standard anterolateral and anteromedial portals are created. A comprehensive intra-articular exploration is conducted. Any associated cartilaginous or meniscal lesions are managed during this phase. The ACL remnant is excised, and the ACL femoral footprint is cleared to facilitate anatomic tunnel placement.

### Step 3: Tunnel Preparation and Graft Passage

**Fig 6:**
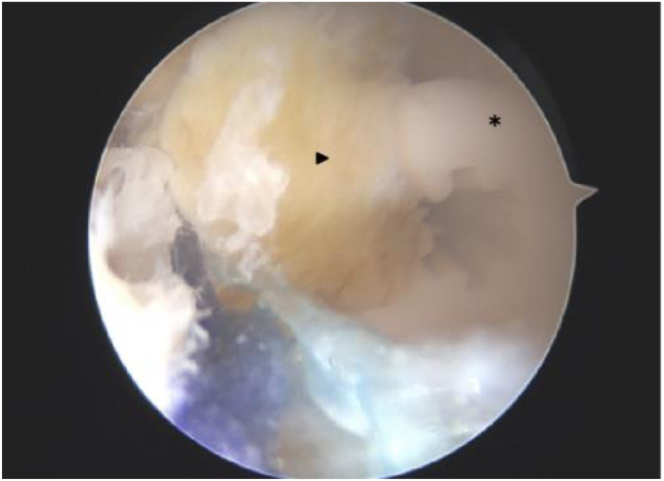
Arthroscopic view of tibial tunnel graft fixation using an interference screw. The arthroscope positioned within the tibial tunnel reveals the interference screw placement. The screw (asterisk) is seated flush with the distal apex of the bone block (arrow), maximizing engagement of the screw’s entire length to enhance graft fixation strength.

Femoral and tibial tunnels are created using an outside-in technique. Guide pins are placed under direct arthroscopic vision on the ACL footprint. The femoral tunnel reaches the lateral femoral cortex 5 mm posterior and proximal to the lateral epicondyle.^27^ Tunnels are drilled to a diameter that matches the graft size. The graft is passed from the tibial to the femoral tunnels. Femoral fixation is achieved using a 20-mm-long FastThread interference screw (Arthrex). On the tibial side, the graft is fixed with a 30-mm-long FastThread screw, the position of which is controlled using the arthroscope (Fig 6). An additional tibial fixation is performed in the medial tibial cortex using a 4.75-mm SwiveLock anchor (Arthrex), securing the sutures passed in the graft.

### Step 4: ALL Reconstruction and Capsular Reinforcement

**Fig 7:**
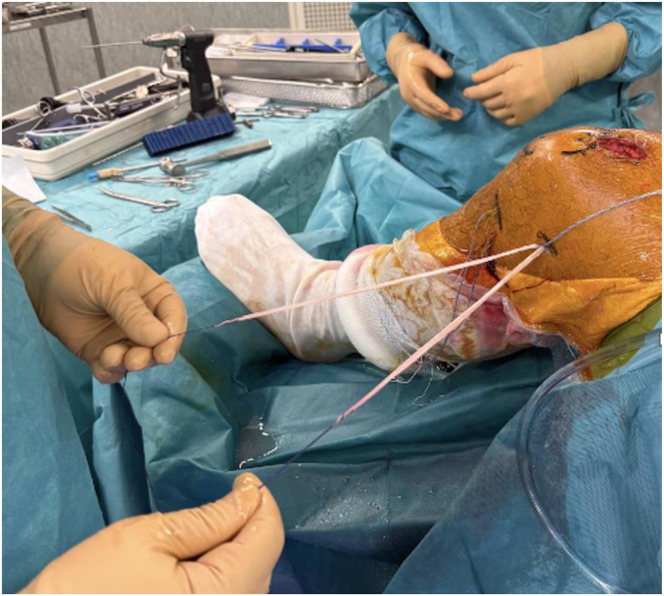
Lateral view of a left knee after anterior cruciate ligament graft fixation. The 2 strands of the rectus femoris exit the femoral tunnel, from which a 4-strand anterolateral ligament reconstruction can be performed.

**Fig 8:**
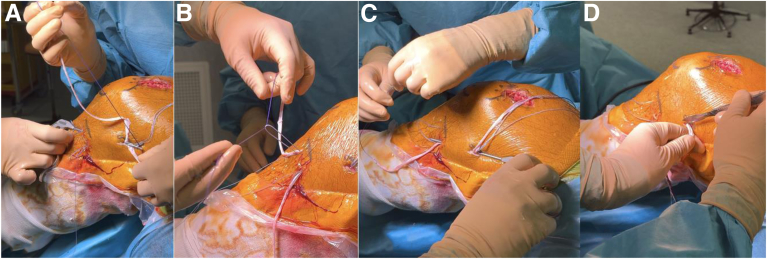
Anterolateral ligament reconstruction. Lateral view of the left knee in flexion; 2 tibial skin incisions are made 1 and 3 cm posterior to Gerdy’s tubercle and 1 cm distal to the lateral tibiofemoral joint line. Two knotless self-locking anchors are placed in the tibia through each incision. A first strand of the rectus femoris (RF) graft is retrieved underneath the iliotibial band from the femoral to anterolateral tibial incision (A), passed through the anchor suture loop (B), and fixed securely with the knee in full extension and neutral rotation. The free end of the same strand is then retrieved back to the femoral incision, still beneath the iliotibial band (C). The same procedure is repeated for the second RF strand to the posterior tibial anchor. The 2 strands are then fixed to the femur using the traction sutures from the anterior cruciate ligament graft, with the knee in full extension and neutral rotation. Finally, the excess graft is cut (D).

ALL reconstruction is performed using the double-strand rectus femoris graft (Fig 7). Two 1-cm skin incisions are made at the lateral tibial plateau, posterior to Gerdy’s tubercle and 1 cm distal to the joint line according to the principles of Sonnery-Cottet et al.^28^ Two FiberTak Knotless anchors (Arthrex) are placed in the tibial cortex. Each limb of the rectus femoris graft is passed beneath the iliotibial band, one directed toward each anchor, and fixed securely. Proximally, the 2 limbs are retrieved toward the femoral tunnel and sutured to the traction threads of the ACL graft exiting the femoral tunnel. Fixation is performed with the knee in full extension and neutral rotation, enabling ALL reconstruction with a 4-strand construct (Fig 8).

### Step 5: Closure and Postoperative Management

**Fig 9:**
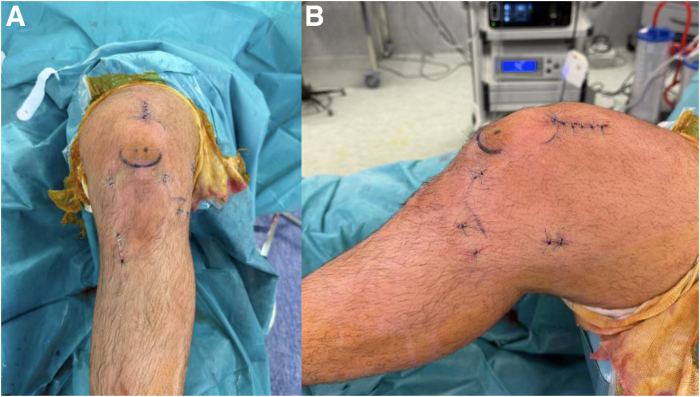
Final aspect after skin closure: anterior view (A) and lateral view (B).

A periarticular injection of 20 mL levobupivacaine (2.5 mg/mL) is administered for analgesia. Layered closure is performed: fascia and deep tissue with absorbable sutures, as well as skin with nonabsorbable monofilament sutures (Fig 9). A standard ACL rehabilitation protocol is initiated postoperatively, focusing on vastus medialis activation to limit atherogenic muscle inhibition.^29^

## Discussion

Recent interest has focused on the RF component of the quadriceps, which provides longitudinally oriented fibers with favorable anatomic length and diameter for graft use.^20^, ^21^, ^22^, ^23^ While promising, these approaches relied solely on soft tissue fixation excluding bone-to-bone fixation that the extensor mechanism autografts can offer. Moreover, unlike QT, hamstring tendon, or bone–patellar tendon–bone autografts, no biomechanical studies have yet shown that the RF autograft shows mechanical properties suitable for ACL reconstruction.

Our technique builds on these foundations by introducing a continuous graft composed of a patellar bone block, an adjacent segment of quadriceps tendon, and a long strand of rectus femoris. The rationale for this design is multifactorial. First, the addition of a bone block addresses the critical need for secure tibial fixation and integration in revision cases where tunnel widening, osteolysis, or previous hardware compromises graft fixation and later integration. Bone-to-bone healing has shown superior fixation strength and faster incorporation in both primary and revision settings compared with soft tissue to bone.^10^^,^^11^^,^^24^

Second, the extension of the graft beyond the rectus femoris to include a portion of the quadriceps tendon ensures the use of an autograft, showing strong clinical and biomechanical data for ACL reconstruction. In patients requiring additional rotational control, the longitudinal splitting of the RF portion enables a double-bundle configuration, permitting simultaneous reconstruction of the ACL and the ALL. This provides a single-donor solution, avoiding the need for additional harvest sites or allografts.

Third, the double construct made of 2 bundles each may increase the biomechanical resistance of the ALL construct. This falls within the strategy to “do more” in the setting of revision surgery if the ALL had already been reconstructed and when no risk factors for iterative ACL rupture (e.g., excessive tibial slope, meniscal lesion, tunnel mispositioning) can be addressed during the operation. Finally, a continuous graft offers secondary femoral fixation of the ACL graft during the ALL reconstruction.

Compared to the isolated RF techniques described by Huber et al.^20^^,^^21^ and Rego et al.,^22^ this technique may offer greater structural robustness and enhanced tibial and femoral fixation.

This technique, however, has substantial drawbacks. The amount of soft tissue harvested often exceeds the length necessary for ACL and combined ALL reconstruction. The use of a stripper-cutter when harvesting the RF strand can rule out this issue and offers a customized harvest. Yet, there is lack clinical data concerning complications and extensor strength deficit. While early anatomic and clinical results are promising, further studies are needed to evaluate long-term outcomes in terms of graft survival, functional recovery, and quadriceps strength (Table 1).

**Table 1:** Pearls and Pitfalls

Step	Pearls	Pitfalls
1. Quadriceps tendon harvest	Include a patellar bone block for secure bone-to-bone fixation	Insufficient bone block may compromise fixation strength
2. Preservation of the deep layer	Maintain integrity of the deep tendon layer to avoid capsule violation	Capsular effraction leading to joint communication
3. RF tendon identification	Use blunt O’Shaughnessy dissection to clearly isolate the RF	Aggressive dissection may damage surrounding fibers
4-5. RF tendon harvest	Harvest with knee flexed 20°, with the guide stripper toward the AIIS	Inadequate knee flexion may shorten graft or cause rupture
6. Graft preparation	Split RF into 2 strands for modular use	Poor separation weakens strands and construct
7. Femoral tunnel drilling	Position entry point 5 mm postero-proximal to the lateral epicondyle	Malposition compromises graft isometry and stability
8. ALL reconstruction	Use knotless suture anchor for secure fixation	Anchor misplacement reduces rotational control
9. Fixation	Fix graft with knee in full extension and neutral rotation	Fixation in flexion/rotation may cause overconstraint

AIIS, anterior inferior iliac spine; ALL, anterolateral ligament; RF, rectus femoris.

In summary, the rectus femoris–bone–quadriceps composite graft represents a versatile and anatomically favorable solution for ACL reconstruction. It offers a balanced approach between soft tissue adaptability and bone-to-bone fixation, and its modular design allows for combined intra- and extra-articular reconstructions from a single autologous source. The bone–quadriceps tendon–rectus femoris continuous autograft represents a reliable and technically adaptable option for ACL reconstruction, particularly well suited to revision cases (Table 2).

**Table 2:** Advantages and Limitations

Advantages	Limitations
Single autograft harvest site for combined ACL and ALL reconstruction	Lack of data on quadriceps muscle morbidity after RF harvest
Improved fixation and integration using a patellar block in the tibial tunnel	Risk of patellar fracture
Continuous autograft enables a single femoral fixation for both ACL and ALL grafts	Risk of posterior femoral wall blowout during tunnel creation or graft fixation
Sufficient graft volume and length	Graft volume and length often exceeding requirements
Onlay tibial ALL graft fixation: simple and bone-preserving technique	Onlay tibial ALL graft fixation: possible reduced tendon-to-bone healing compared to inlay fixation

ACL, anterior cruciate ligament; ALL, anterolateral ligament; RF, rectus femoris.

## Disclosures

The authors declare the following financial interests/personal relationships which may be considered as potential competing interests: M.V. is a consultant or advisor for Arthrex. O.M. is a consultant or advisor for Stryker. All other authors (V.G., P.E., G.B., G.P., H.C.) declare that they have no known competing financial interests or personal relationships that could have appeared to influence the work reported in this paper.

## Supplementary Data

Video 1 We describe a surgical technique for the simultaneous reconstruction of the anterior cruciate ligament and the anterolateral ligament (ALL) using a continuous autograft composed of a patellar bone block, the quadriceps tendon, and the rectus femoris aponeurosis. A 4-mm midline skin incision is made to expose the quadriceps tendon and the proximal aspect of the patella. A patellar bone block measuring 1 cm in length and 2 cm in width is delineated on the patella. Two 2-mm diameter holes are drilled into the center of the bone block, which is subsequently harvested using an oscillating saw. Two No. 2 traction sutures are passed through the bone block to facilitate graft handling and traction during both harvest and fixation. The sutures can be used later to perform a secondary tibial fixation. Dissection is then carried out proximally to harvest a partial quadriceps tendon autograft approximately 1 cm in width while preserving its deep layer. Once 8 cm of the tendon has been harvested, the rectus femoris (RF) aponeurotic layer is identified at the proximal end and harvested using an open stripper. At this stage, a stripper-cutter device may be preferred to harvest the desired length of RF aponeurosis. The harvested graft is then cleaned of residual muscle fibers, and the RF aponeurosis is split into 2 separate strands. Traction sutures are applied to each strand, and the graft is calibrated to determine the appropriate diameter of the femoral and tibial tunnels. For ALL reconstruction, 2 knotless suture anchors are placed on the tibia approximately 1 cm distal to the lateral joint line and posterior to Gerdy’s tubercle to perform a double-strand ALL reconstruction. Arthroscopic examination of the knee is performed in a standard manner. Femoral and tibial tunnels are drilled using an outside-in technique, followed by the insertion of a shuttle suture through the tunnels. A guidewire is introduced into the femoral tunnel, and the graft is pulled into the joint from distal to proximal with the patellar bone block in the tibial tunnel. The quadriceps tendon autograft is secured first in the femoral tunnel using a size-to-size interference screw, the position of which is controlled using the arthroscope. Tibial fixation is achieved with another interference screw, which is placed flush with the bone block to maximize fixation strength. Appropriate graft tension is confirmed. A shuttle suture is used to prepare the self-locking loop of each tibial anchor, which will secure the 2 RF strands for ALL reconstruction. The RF strands are passed from proximal to distal beneath the iliotibial band using low-profile arthroscopic forceps. Each strand is passed through the anchor loop and secured with the knee in full extension and neutral rotation by tensioning the self-locking mechanism. The 2 strands are redirected proximally under the iliotibial band toward the femoral incision and fixed using the traction sutures previously placed on the quadriceps tendon graft, still with the knee in full extension. This completes a 4-strand construct for ALL reconstruction. If necessary, the sutures from the patellar bone block may be anchored to the tibia to reinforce the tibial fixation. At 1 month postsurgery, the patient recovered complete knee extension, good quadriceps activation, and more than 120° of flexion.
